# Impact of Wetting–Drying Cycles on the Mechanical Properties and Microstructure of Wood Waste–Gypsum Composites

**DOI:** 10.3390/ma12111829

**Published:** 2019-06-05

**Authors:** Manuel Alejandro Pedreño-Rojas, María Jesús Morales-Conde, Paloma Rubio-de-Hita, Filomena Pérez-Gálvez

**Affiliations:** Departamento de Construcciones Arquitectónicas 1, Escuela Técnica Superior de Arquitectura, Universidad de Sevilla, Avenida Reina Mercedes, n_ 2, 41012 Sevilla, Spain; mmorales@us.es (M.J.M.-C.); palomarubio@us.es (P.R.-d.-H.); fipergal@us.es (F.P.-G.)

**Keywords:** gypsum plasters, construction and demolition wastes, accelerated climatic aging, mechanical performance, ultrasonic velocity, SEM

## Abstract

Large amounts of wood waste are generated each year in the world. In an attempt to identify a good recovery option for those residues, wood waste from construction and demolition works were used as raw materials in gypsum plasters. However, wood is a biodegradable material which implies that the products or materials that contain it are susceptible to suffering an important deterioration, due to exposure in certain environments. For that reason, the aim of this work was to simulate the effects that, in the long term, the atmospheric exposure of wood waste–gypsum composites would have. To do that, the plasters were subjected to 5, 10, and 15 wetting–drying cycles in a climatic chamber. In this study, the density, flexural and compressive strength, and ultrasonic velocity of these composites were determined by the influence of the aging process on their mechanical properties. Furthermore, in order to detect changes on their internal structure, scanning electron microscopy tests (SEM) were used. The results showed that they were suitable to be used as indoor coverings of buildings. However, a treatment to reduce the moisture absorption of the wood waste must be studied if mixtures with high percentages of wood shavings (WS20) are used in wet rooms.

## 1. Introduction

Correct waste management is one of the main lines of work established by the European Commission within commitments of Horizon 2020 [1]. In particular, the amount of wood waste varies significantly, depending on the typical structural/construction typology of the country or region under analysis. It is especially significant in northern countries, such as Norway, where it means 10.12% of the total construction and demolition wastes (CDWs) generated. In general, in Europe, wood waste represents an average of 2.6% of the total waste created by the building sector [2].

Mália et al. found that, when only residential building demolition works wastes were analyzed, the percentage of wood waste increased significantly, reaching 37.9% of the total CDW generated in those type of buildings [3]. For all these reasons, and although wood is a highly renewable and regenerative material, it is necessary to propose correct management of this waste.

Although cement particleboards are the most extended use for wood waste in the building sector, many researchers have developed new construction composites by using them as raw material. Most of those studies analyzed the use of the wood waste to create a lightweight concrete [4,5,6]. Furthermore, the development of wood-cement composites has also been studied, obtaining an improvement of their thermal properties [7,8]. Meanwhile, in 2016, Morales-Conde et al., to identify a method for wood waste generated in rehabilitation works, tested new gypsum composites with wood particles (sawdust and wood shavings). A lighter material with improved thermal properties was obtained [9]. Those composites were subsequently used to develop prefabricated gypsum plates for false ceilings with improved thermal and acoustic properties [10].

On the other hand, several works have studied the influence of an accelerated climate aging process on diverse composite materials, trying to simulate the effects it would have on the material after its exposure in different climatic conditions. Sharman was one of the first who tested wood–fiber–cement materials, previously submitted to an autoclaved treatment, to several accelerated ageing processes [11]. After that, Soroushian et al. studied the durability of wood–cement composites submitted to wetting–drying and freezing–thawing cycles [12]. Wei and Meyer analyzed the effects of various accelerated deterioration procedures on the physical and mechanical properties of cement mortars reinforced with sisal fiber. After being subjected to 5, 15, and 30 cycles of wetting and drying, it was observed that there was a significant decrease in the resistant capacities of these compounds [13,14,15]. Sisal fiber cement degradation and aging mechanisms have also been analyzed by other researchers. They observed that the degradation process of the fibers occurred very quickly, obtaining a large decrease on the flexural strength of the composites after 10 cycles of wetting–drying [16]. The durability of wood waste composites was researched by Coatanlem et al. The concrete samples were exposed in humid and dry environments, and their flexural and compressive strength after the aging process were tested. They also used a sodium silicate solution treatment on the wood chippings, in order to improve the bonding at the wood–cement interface [17]. The effects of wetting–drying cycles on the microstructure and mechanical behavior of concrete containing rice husk ash as a partial substitute of cement were also studied [18].

As was observed, most of the works that analyze the influence of aging cycles in new materials focus on concrete and cement-based compounds [19,20,21,22,23]. However, Belayachi et al. analyzed the long-term serviceability of gypsum plaster–straw insulation material [24]. To simulate the external conditions, the materials were subjected to freezing–thawing and wetting–drying aging tests. In this case, it was affirmed that the greatest changes in relation to mechanical resistance and thermal behavior, with respect to the non-aged material, took place when the samples were subjected to wetting–drying cycles, while in the case of freezing–thawing cycles, this difference was minimal. Furthermore, the effects of wetting–drying cycles on the mechanical performance and microstructure of gypseous soils have also been studied, finding that the biggest strength loss was achieved in soils with higher gypsum contents [25].

Although there are some published studies on gypsum-based materials containing wood waste, no previous experience has been found related to the study of the effects of accelerated climatic aging in those composites.

Wood is a biodegradable material, which implies that the products or materials that contain it are susceptible to suffer an important deterioration, due to its exposure in certain environments. For that reason, the aim of this work was to simulate the effects that, in the long term, the atmospheric exposure of these wood waste–gypsum materials [9,10] would have. To do that, wood waste–gypsum plasters were subjected to 5, 10, and 15 wetting–drying cycles in a climatic chamber. In this study, the density, flexural and compressive strength, and ultrasonic velocity of these composites were determined, analyzing the influence of the aging process on their mechanical properties. Furthermore, in order to detect changes on their internal structure, scanning electron microscopy tests (SEM) were used.

## 2. Materials and Methods

### 2.1. Materials

#### 2.1.1. Gypsum

For the generation of the new wood waste–gypsum composite materials, traditional commercial gypsum for construction (type B1), according to the UNE-EN 13279-1 standard [26], was used. Their characteristics are shown in Table 1.

#### 2.1.2. Wood Waste

**Figure 1:**
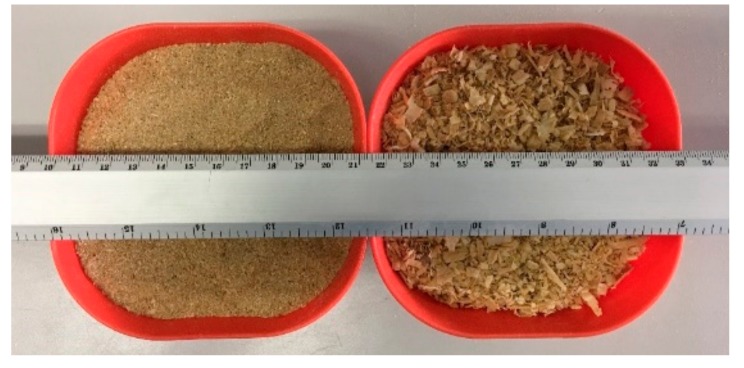
Sawdust and wood shavings used as raw material in the development of the new gypsum plasters.

Wood shavings (WS) and sawdust (S) were used as raw materials in the plasters (Figure 1). The wood waste used for this work came from old *Pinus* wooden slabs (timber beams and joists), that were replaced or demolished in rehabilitation works.

**Figure 2:**
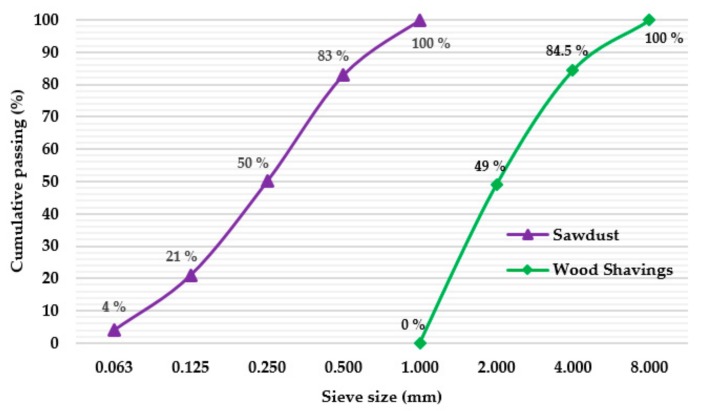
Particle size distribution of wood waste used as raw material.

When the material was received, the wood waste was submitted to a surface cleaning process, using wire brushes, to eliminate any possible sand/dirt adhered to the material. After that, the wooden pieces were crushed using a TELEMECANIQUE crusher. To obtain the sawdust (0.063–1.000 mm) and wood shavings (1.000–8.000 mm), different PROETI sieves were used, as shown in Figure 2.

**Figure 3:**
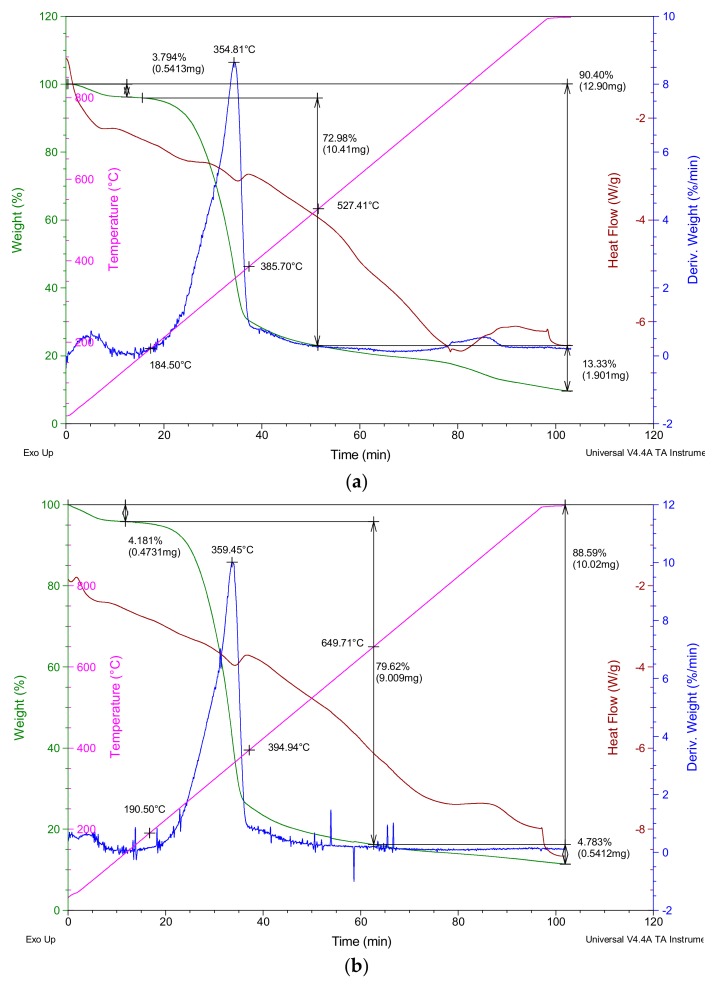
Thermogravimetric analysis (TGA) results. (**a**) Sawdust; (**b**) wood shavings.

Once the wood residue was classified into chips and sawdust, an exhaustive characterization of both was carried out. First, to obtain the moisture content of the material, the wastes were placed in an oven until constant mass (18 h at 50 °C). A relative humidity of 5.8% for sawdust and 6.3% for wood shavings was obtained. Subsequently, the true density of both materials was obtained by helium pycnometry, using a QUANTA CHROME stereopycnometer (Boynton Beach, FL, USA). The results showed that sawdust had a true density of 1.2 g/cm^3^ and wood shavings had one of 1.5 g/cm^3^. Finally, a thermogravimetric analysis (TGA) was carried out using a TA SDT Q600 equipment (New Castle, DE, USA), and the results are shown in Figure 3.

### 2.2. Sample Preparation

The determination of the water/gypsum ratio of the composites was carried out following the guidelines established by the consistency tests in regulations [27]. These tests revealed that, for samples with a wood containing equal to or lower than 10% by weight of gypsum, the optimum ratio to be used was 0.55. However, in the samples with 20% wood waste, this ratio increased to 0.80.

Table 2 contains the composition of the seven mixtures developed in this work:

Following these proportions, 12 prismatic specimens of 40 × 40 × 160 mm^3^ [27] for each mixture were elaborated, using a PROETISA mortar mixer (Sevilla, Spain). After that, according to the standard [27], the samples were placed in a curing chamber for seven days at 24 °C, with a relative humidity of 50 ± 1%. Later, they were dried in an oven at 40 ± 2 °C to constant mass.

### 2.3. Accelerated Aging Procedures

As is well known, gypsum plasters are mainly used as indoor coverings of buildings. For that reason, and in order to simulate the indoor conditions of gypsum plasters in wet rooms (bathrooms, kitchens, etc.), wetting–drying tests were carried out on the gypsum specimens after 7 days in a curing chamber.

The wetting–drying cycles were performed on the wood waste–gypsum plaster for a duration of 168 h (7 days) using the procedure described on UNE-EN 9142 [28], as shown in Table 3.

**Figure 4:**
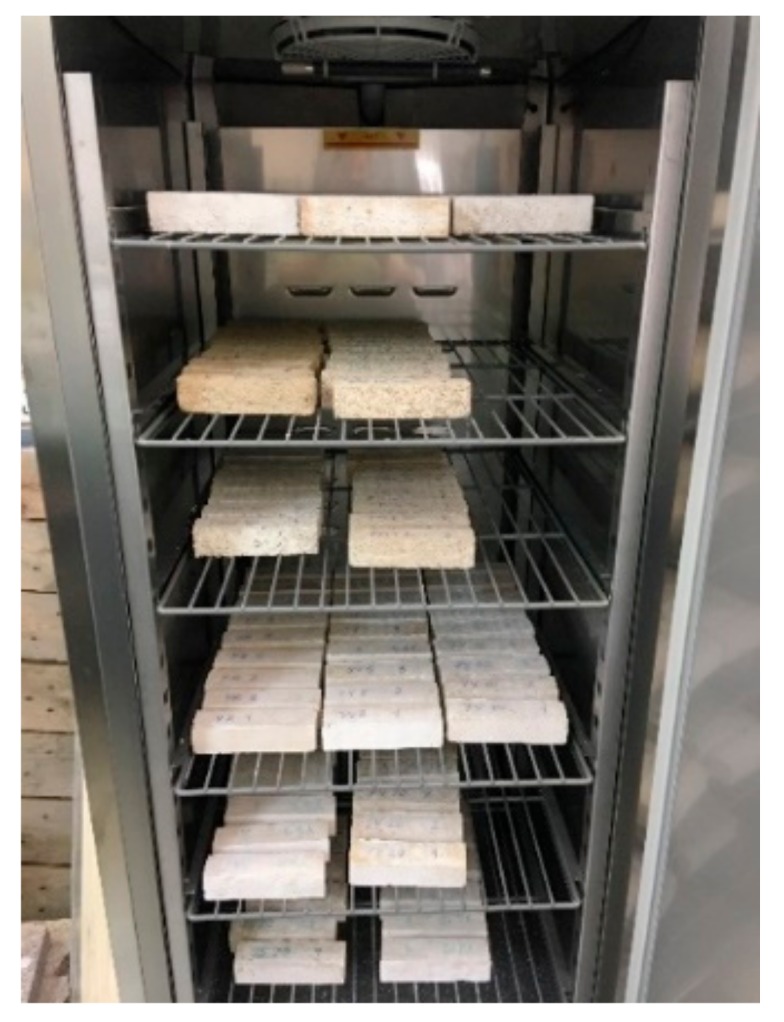
Samples during the wetting–drying cycles in the curing chamber.

Samples were exposed to 5, 10, and 15 wetting–drying cycles in order to amplify the aggressive treatment. The wetting–drying cycles were conducted in a CCI CLIMATICA STD curing chamber (Universidad de Sevilla, Sevilla, Spain) (Figure 4). For each cycle, the samples were weighed before and after drying in order to monitor water content.

### 2.4. Test Methods

Wetting–drying cycles were carried out in batches of 5, 10, and 15 repetitions. To conduct the experimental program, 12 test samples of each mixture were elaborated (3 samples per repetition batch and 3 samples to be broken before the cycles). Once each cycle had been completed, a visual examination of the specimens was carried out to establish a first approximation of the possible damages produced in them (cracks, surface degradation, spots of mildew, etc.). Then, the samples were weighed, analyzing the losses or gains of weight at the end of each wetting–drying cycle.

In order to obtain the mechanical properties of the plasters, the flexural and compressive strength of the specimens were determined at the end of each batch of repetitions. To justify the results obtained, an ultrasound measurement and a scanning electron microscopy (SEM) analysis of the samples were carried out.

**Figure 5:**
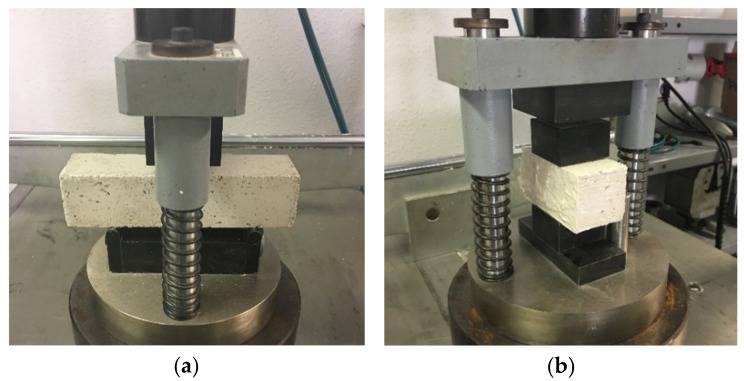
Samples during the mechanical tests. (**a**) Flexural strength; (**b**) compressive strength.

The following test procedures were used to obtain the mechanical behavior of the new plasters:

- *Flexural strength:* The three points bending test (Figure 5a) was used to determine the flexural strength of the new composites, according to standard UNE-EN 13279-2 [27]. To obtain the bending strength of the plasters, three measurements were conducted for each mixture, obtaining the flexural strength of the materials as the mean value of those three samples.
- *Compressive strength:* The 6 half-pieces obtained from the flexural strength test were used to obtain the compressive strength of the new plasters, according to standard UNE-EN 13279-2 [27]. During these test, a central progressive load was applied to the specimen, obtaining the compressive breaking load value of each sample (Figure 5b). The mean value of the six tested samples was obtained to determine the compressive strength of each mixture.
  A statistical analysis of the resistance tests results was also carried out. Both mechanical tests were performed using the SUZPECAR multi-test machine (Universidad de Sevilla, Sevilla, Spain), a press with a 20-ton load capacity and a precision that varies according to the control type. In the control per round, the precision is 0.01 mm/min, whereas in load control the precision is 0.1 kg/s.
- *Ultrasonic velocity:* Before rupture, the specimens were submitted to an ultrasonic velocity test, following the procedure described by UNE-EN 12504-4 standard [29]. The ultrasonic velocity is expressed as the relation between the length of the trajectory and the time elapsed, between the beginning of the pulse wave generated in the emitter probe, and the beginning of the wave on its arrival to the receiver probe. This technique is helpful in the determination of the homogeneity of a material, being related to the density, porosity, and elastic properties of the plasters. The PUNDILAB (NEURTEK) ultrasonic machine (Gipuzkoa, Spain) was used for the tests. The accuracy was ±0.1 μs. The emission frequency of the machine used was 50 KHz.
- *Scanning electron microscopy (SEM):* The FEI TENEO field emission scanning electron microscope (Hillsboro, OR, USA) was used in this test. To achieve adequate sample conductivity, the EDWARDS sputter six scancoat metallizer was applied to a superficial pattering of gold.

## 3. Results and Discussion

As a summary, all the results obtained from the tests performed and their measurement variability (CoV) are compiled in Table 4. 

### 3.1. Visual Examination of the Specimens

At the end of each round of wetting–drying cycles, a visual inspection of the samples was carried out to locate any possible damage caused by the accelerated aging of the materials.

**Figure 6:**
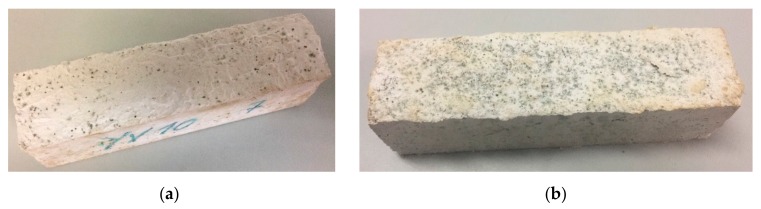
Small spots of mildew in wood shavings plasters after 15 wetting–drying cycles. (**a**) WS10; (**b**) WS20.

**Figure 7:**
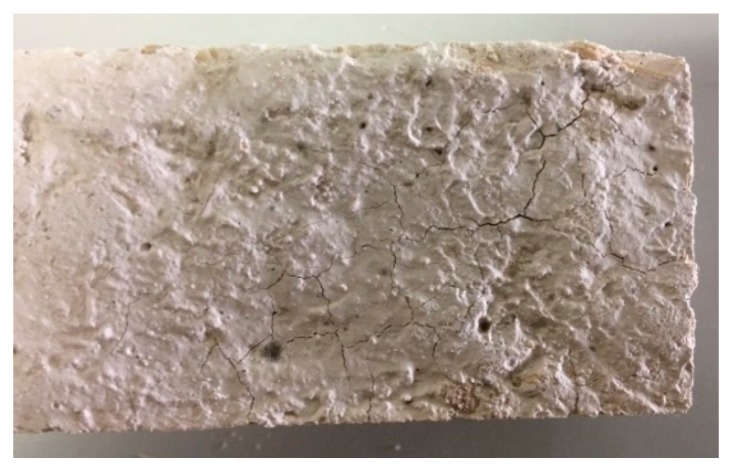
Small cracks in WS20 samples after 15 wetting–drying cycles.

After the inspection executed at the end of the first 5 and 10 cycles, no damage was detected in the samples. However, after completion of the whole process (15 cycles), damage was seen in the samples with wood shavings with a higher percentage of waste. Specifically, WS10 and WS20 showed small spots of mildew in the surface of the sample as shown in Figure 6. This proliferation was far more significant in the mixture with 20% waste (WS20). Furthermore, small cracks and surface degradation appeared in the surface of the WS20 samples after 15 wetting–drying cycles (Figure 7). Finally, it is important to note that no surface damage was observed in any of the mixtures that used sawdust as raw material throughout the test plan.

Due to its high hygroscopicity and the abrupt humidity changes during the tests, the wood was susceptible to the proliferation of fungi (mildew). These, together with the expansion and retraction of the sample due to the presence of humidity, are the cause of the damage seen in the visual examination of the mixtures.

### 3.2. Density

**Figure 8:**
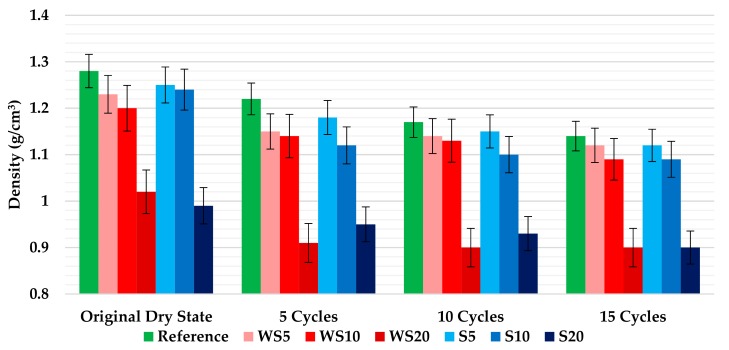
Density of the plasters after 0, 5, 10, and 15 wetting–drying cycles.

Figure 8 shows the density values of the mixtures at the end of each batch of cycle repetitions. It was observed that, for all the mixtures, the increase in the percentage of wood waste added to the plaster led to a decrease in the density in relation to the reference material. That drop in density was most pronounced in mixtures with wood waste aggregate at 20%, an important drop in relation to the value achieved for composites with 10% of waste. The reduction obtained was in accordance with the results obtained previously by Morales-Conde et al. [9] and by Dai and Fan [30], who also used wood waste for the development of gypsum plasters. On the other hand, it could be observed that, for all the plasters, the increase in the number of wetting–drying cycles was linked to a decrease in density in relation to those that had not been submitted to the aging process (0 cycles). The biggest drop was achieved for the WS20 samples, reducing the density of the plaster by 11.8% after 15 wetting–drying cycles. This reduction was due to loss of mass, caused mainly by the small disintegration process, that was suffered by the composites with the highest percentage of waste, when they were subjected to the wetting–drying cycles. Finally, it must be said that other studies from the literature also achieved an important reduction in the density of the materials when they were submitted to wetting–drying cycles [13,17,24].

### 3.3. Moisture Content

**Figure 9:**
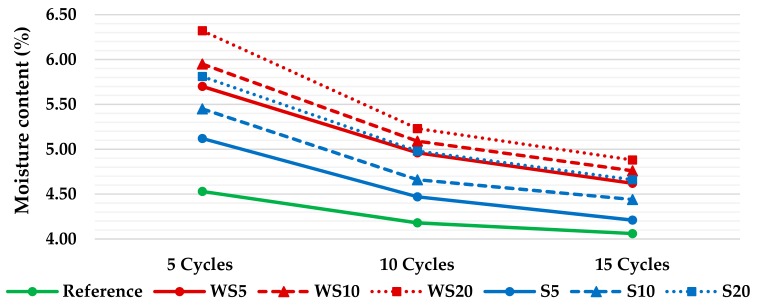
Moisture content evolution of the plasters during the cycles.

Once the specimens were submitted to each batch of cycle’s repetitions, they were weighted and dried to the absolute dry state. Following that procedure, the moisture content of the mixtures after 5, 10, and 15 cycles was obtained as shown in Figure 9. As can be seen, the moisture content of the samples was reduced when the number of wetting–drying cycles increased. This fall was more significant for samples with a higher wood waste content (mainly WS20). In addition, the most important decrease occurred during the first cycle, and was almost stabilized when the 15th cycle was finished. Those results are in accordance with those obtained for the density of the plasters.

### 3.4. Mechanical Properties

**Figure 10:**
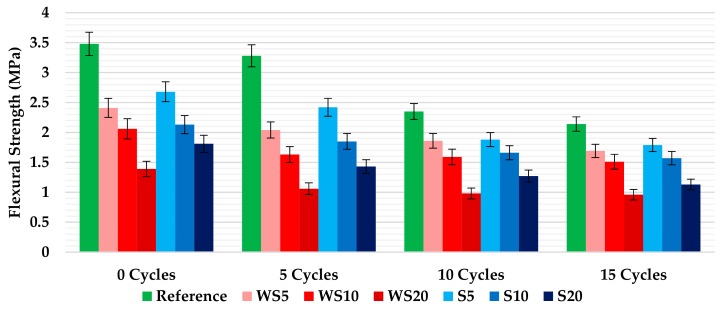
Flexural strength of the plasters after 0, 5, 10, and 15 wetting–drying cycles.

The results obtained for the flexural strength test of the mixtures (and their variability), at the end of each batch of cycle repetitions, are presented in Figure 10. Similar to the density, the increase of the percentage of wood waste added to the plasters meant a decrease in the flexural strength. For all the mixtures, and for the same percentage of waste, mixtures with sawdust showed better mechanical behavior than those with wood shavings. On the other hand, in all cases, the increase in the number of wetting–drying cycles was linked to a significant decrease in the flexural strength of the materials. The fall was more relevant in those composites that contained a lower percentage of wood waste, with the reference material being the one with the biggest drop after 15 cycles (38.9%). It must be considered that, in most of the mixtures, the bending values achieved were higher than the minimum standard requirement (1 MPa) for gypsum plaster, suggesting that is viable to use any of the composites analyzed in construction works [26]. However, the WS20 samples after 10 and 15 wetting–drying cycles (0.98 and 0.96 MPa, respectively) touched the standard requirement without reaching it.

**Figure 11:**
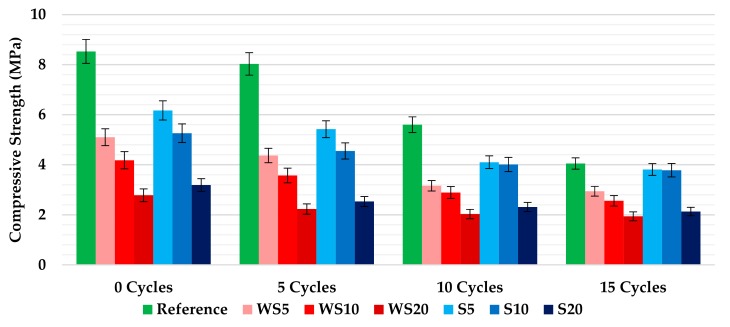
Compressive strength of the plasters after 0, 5, 10, and 15 wetting–drying cycles.

The way in which the new composites behaved when subjected to compression tests is analyzed in Figure 11, which shows the mean strength values, measured in MPa, at the end of each batch of cycle repetitions. As in the other properties analyzed, the increase in the percentage of wood waste added to the mixtures led to a reduction in the materials’ resistance capacities. The specimens with sawdust showed a better compressive strength behavior than those with wood shavings with the same aggregate percentage. On the other hand, in all cases, the increase in the number of wetting–drying cycles was linked to a significant decrease in the compressive strength of the materials. The fall was more relevant in those composites that contained a lower percentage of wood waste, with the reference material being the one with the biggest drop after 15 cycles (52.5%). Most of the results obtained were above the minimum value required for the compressive strength of gypsum plasters (2 MPa), according to the standard [26]. However, the resistance achieved for the WS20 mixture (1.94 MPa), after 15 wetting–drying cycles, was not enough to overcome the standard requirement. The small surface disintegration process that was suffered by the composites with the highest percentage of waste, when they were subjected to the wetting–drying cycles, could be one of the reasons that caused the loss of resistance of the plasters after the aging process.

The variance analysis (ANOVA) for the mechanical properties of the new plasters is shown in Table 5. It shows that the *p*-values associated to each factor (wetting–drying cycles and wood waste addition), and to the interaction of both the flexural and compressive strength results, are lower than the level of significance (α = 0.05). Thus, the null hypotheses of equality of means for both factors could be rejected, both having a statistically significant effect with 95% confidence.

Finally, it must be said that the mechanical properties results can be explained using similar studies from the literature. On the one hand, the decrease in the flexural and compressive strength of the plasters, when the percentage of wood waste added increased, was in accordance with similar previous studies that also used wood shavings and sawdust as raw materials in gypsum plasters [9,10,30]. On the other hand, the behavior presented by the plasters after the wetting–drying tests could also be explained with other papers that conducted different climatic ageing procedures in materials for construction. In all of them, an important drop of both properties’ values (flexural and compressive strength) were achieved after the ageing cycles [13,17,24].

### 3.5. Ultrasonic Velocity

**Figure 12:**
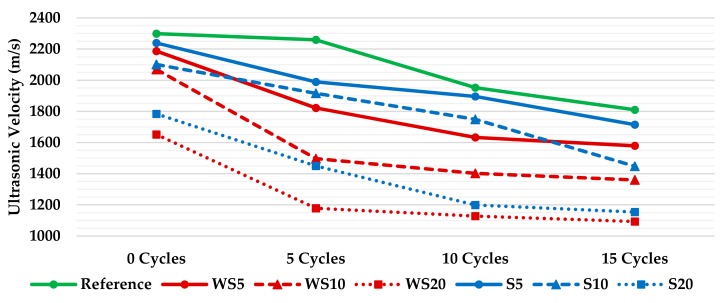
Ultrasonic velocity of the plasters after 0, 5, 10, and 15 wetting–drying cycles.

The ultrasonic velocity depends on the density and elastic properties of a material. So, as can be appreciated in Figure 8, the addition of more wood waste (sawdust and wood shavings), and increased wetting–drying cycles, reduces the density of the plasters. The effects of the wetting–drying cycles in the materials were tested by ultrasonic measurements and the results are shown in Figure 12. The results obtained agreed with the density results, decreasing the ultrasonic velocity as the amount of wood waste in the composites and the number of cycles increased.

An increase of the wood waste percentage and an increase in the number of wetting–drying cycles in the samples was followed by an increase in the porosity of the materials, and a decrease in the density, compactness, and resistance of the plasters.

### 3.6. SEM Analysis

In order to identify the alterations that wetting–drying cycles caused in the microstructure of the plasters, a scanning electron microscopy of the reference composite and mixtures with 20% wood waste (WS20 and S20) was carried out. For that reason, images after 5, 10, and 15 cycles were analyzed.

**Figure 13:**
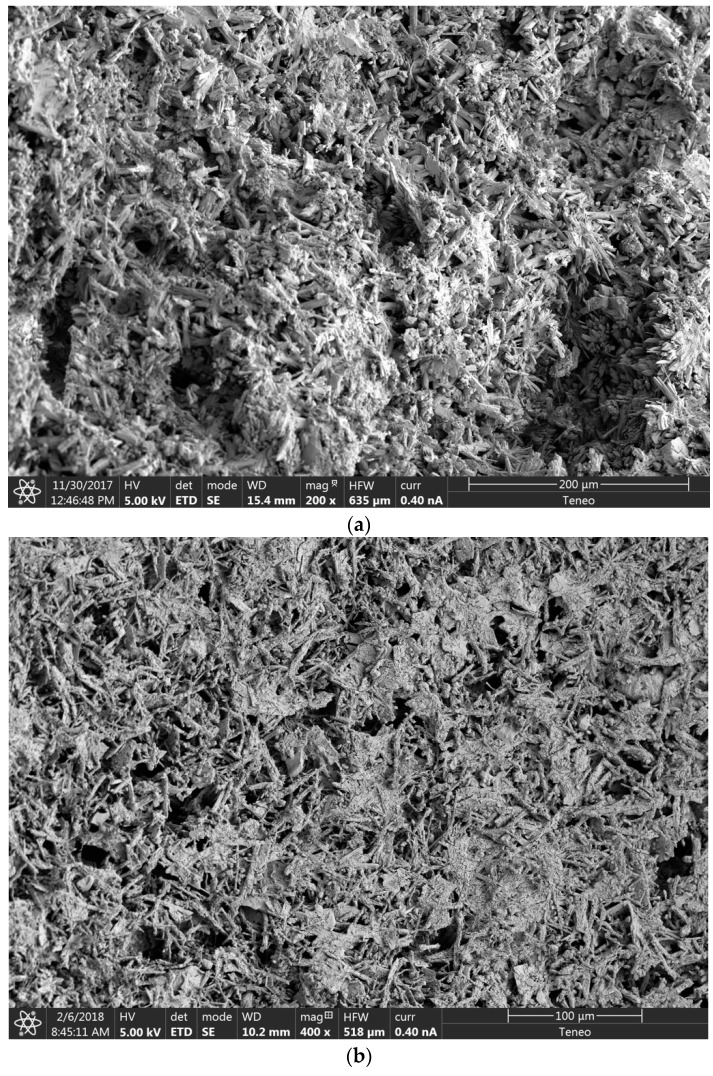

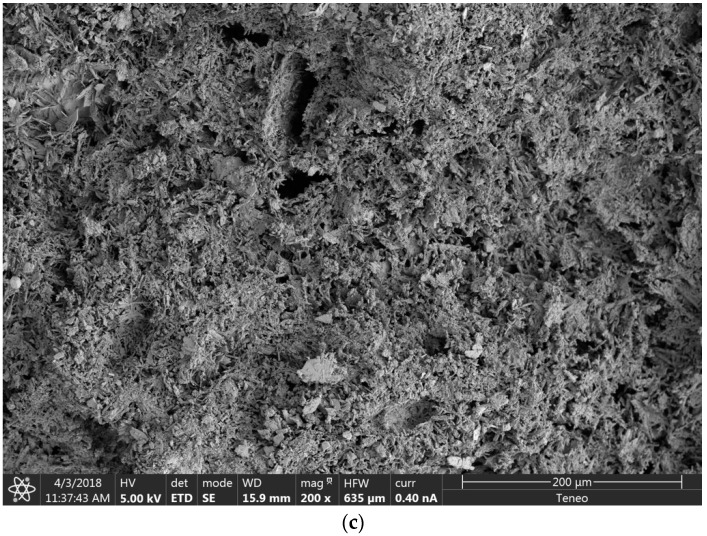
Reference SEM Analysis. (**a**) Five cycles; (**b**) 10 cycles; (**c**) 15 cycles.

The SEM images obtained for the reference plaster (no wood waste addition) are shown in Figure 13. In the images, a progressive modification of the crystalline structure of the material can be observed. After the aging process, an important morphological transformation of the usual crystalline structure of the gypsum plasters was observed. The crystals decreased in size, causing a loss of compactness and strength in the composite.

**Figure 14:**
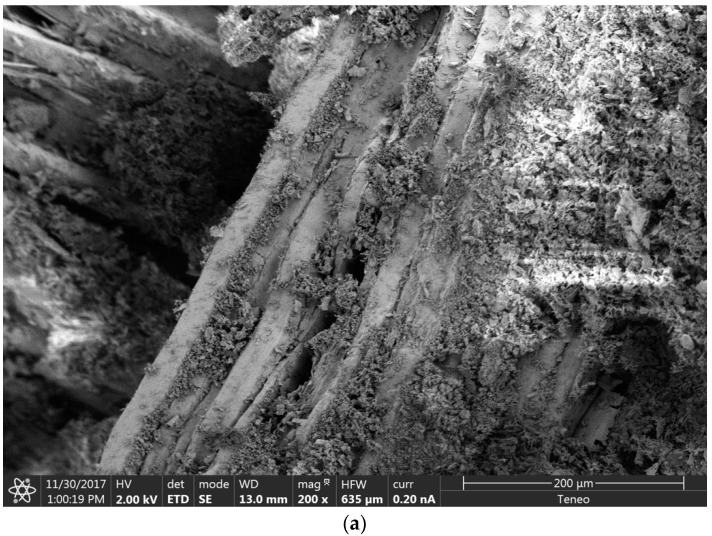

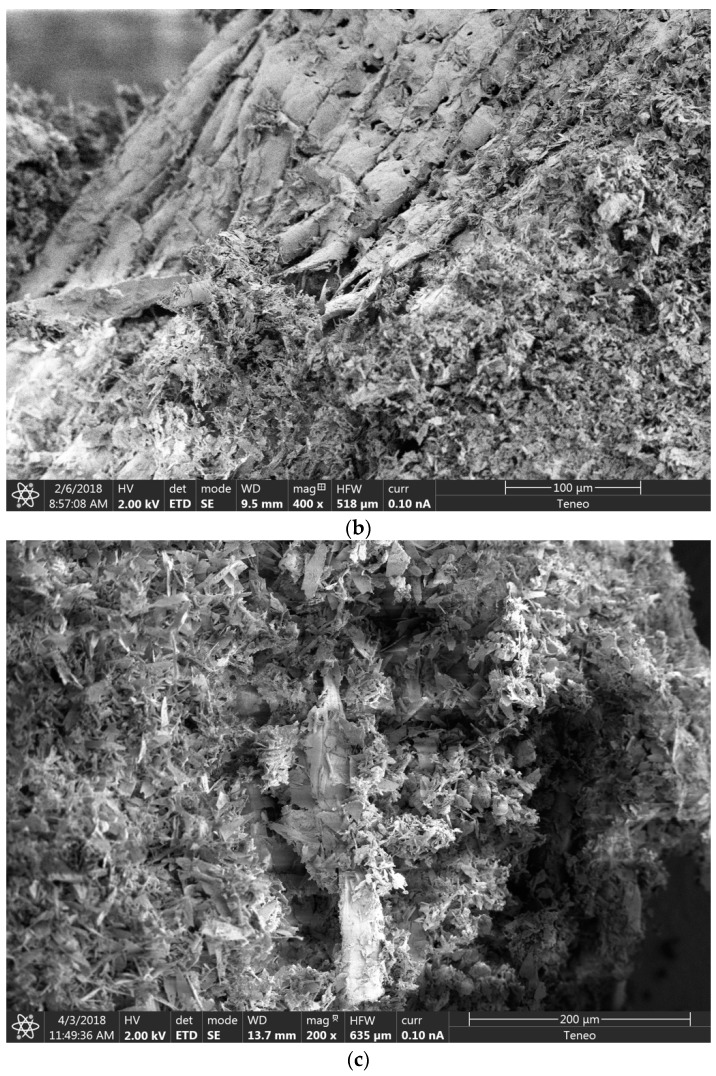
WS20 SEM Analysis. (**a**) Five cycles; (**b**) 10 cycles; (**c**) 15 cycles.

**Figure 15:**
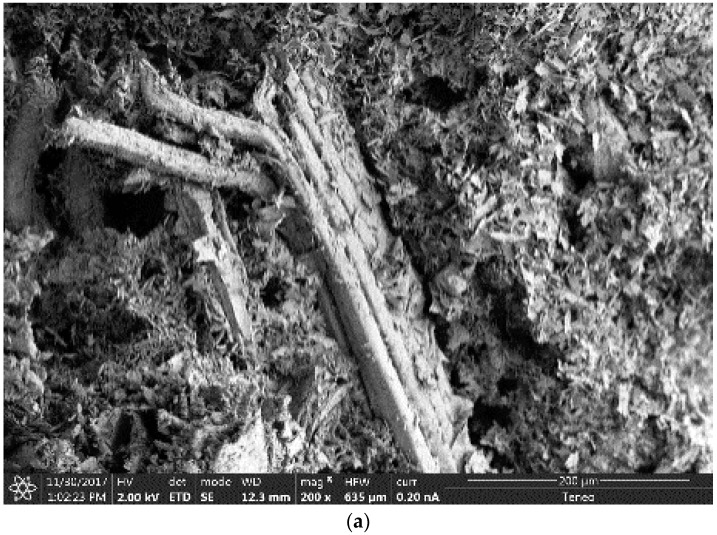

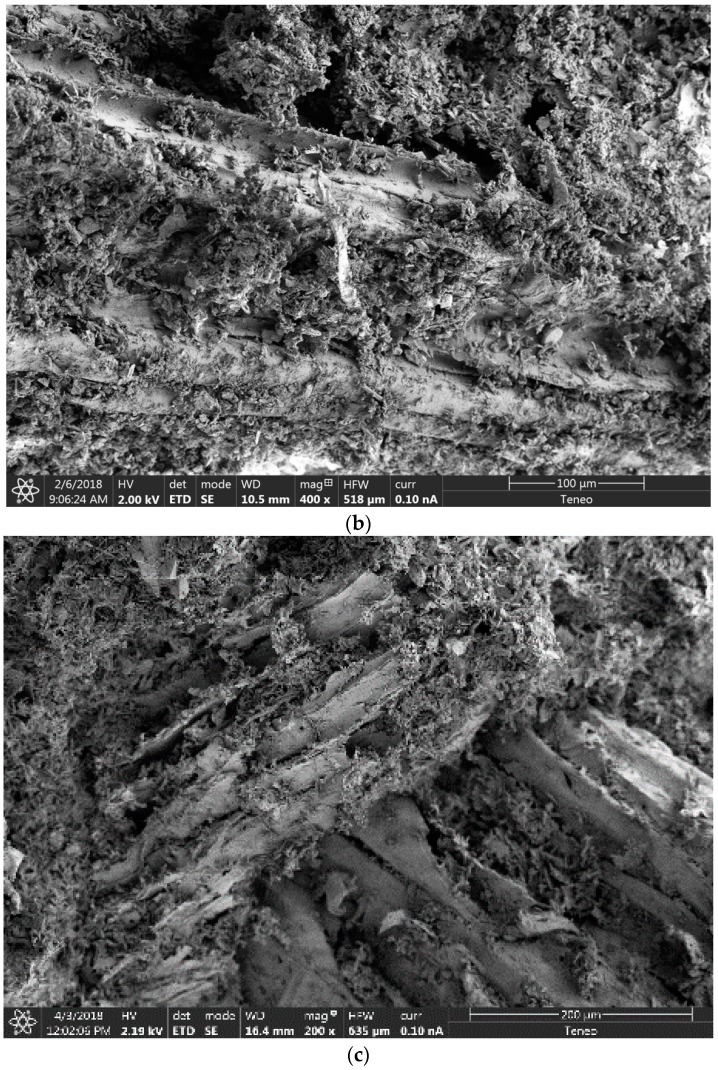
S20 SEM Analysis. (**a**) Five cycles; (**b**) 10 cycles; (**c**) 15 cycles.

SEM images of gypsum plasters with 20% of wood waste after 5, 10, and 15 wetting–drying cycles are presented in Figure 14 (WS20) and Figure 15 (S20). Apart from the transformation in the crystalline structure of the gypsum matrix, the images showed a progressive surface deterioration of the wood waste. Furthermore, the adherence, between the matrix and the wood waste surface, worsened when the composites were subjected to wetting–drying cycles.

According to the results obtained for the mechanical properties of the plasters, it could be said that, despite the surface degradation of the wood waste, the main cause of the resistance loss of the composites was the transformation of the crystalline structure of the gypsum matrix after the ageing process. The wood degradation observed during the ageing process was in accordance with the results reported by Coatanlem et al., when they used wood waste as raw material in concrete [17].

## 4. Conclusions

The experimental work carried out in this article aimed to analyze the influence of wetting–drying cycles on wood waste–gypsum plasters. The results obtained showed that, except for the mixture with the highest percentage of wood shavings (WS20), the composites were perfectly valid for use as building materials, without any special treatment, being able to be exposed both in humid and hot environments without losing their resistant capacities.

Due to the high hygroscopicity of the wood materials and the sudden humidity changes during the tests, small spots of mildew and some cracks were appreciated in the surface of the samples with highest amount of wood shavings (WS10 and WS20) during the visual examination of the specimens after 15 wetting–drying cycles.

Related to the mechanical performance, the same tendency was observed after analyzing the results of the density, flexural, and compressive strength of the composites after subjecting them to the aging process. It could be appreciated that, in all cases, the increase in the percentage of wood waste added to the composite led to a decrease in relation to the reference material. Furthermore, it was observed that, in all cases, an increase in the number of wetting–drying cycles led to a decrease in the mechanical behavior in relation to those that had not been submitted to the aging process (0 cycles). It is important to note that, contrary to expectations, this drop was higher for the reference material (52.5% for compressive strength). Finally, it must be said that all the composites, except for the WS20 mixture, exceeded the minimum values required by the standard for the flexural and compressive strength (1 and 2 MPa, respectively) of gypsum plasters after the aging process.

The ultrasonic velocity test helped to explain the mechanical tests results. An increase of the wood waste percentage and an increase in the number of wetting–drying cycles in the samples was followed by an increase in the porosity of the materials, and a decrease in the density, compactness, mechanical properties, and ultrasound speed of the plasters.

The SEM analysis revealed a transformation in the crystalline structure of the gypsum matrix, a progressive surface deterioration of the wood waste, and a worsening of the adherence between the gypsum matrix and the wood aggregate when the composites were subjected to wetting–drying cycles.

Finally, according to the results obtained for the mechanical properties of the plasters and for the SEM images, it could be said that, despite the surface degradation of the wood waste, the main cause of the resistance loss of the composites was the transformation of the crystalline structure of the gypsum matrix after the ageing process. In summary, the aging tests of the new plasters showed that they were suitable to be used as indoor coverings of buildings.

Despite being a very specific case, the methodology used in this study could be transferred to other research, to predict the behavior of construction materials when they must be exposed to different climatic conditions. Currently, the authors are working on the application of this procedure, along with freeze–thaw cycles, to obtain the durability of wood waste–cement mortars. Furthermore, as future research lines, different treatments (sodium silicate solution ([17]) could be applied to wood waste to improve the durability of plasters with higher contents of wood shavings (WS20) for use in wet rooms.

## Figures and Tables

**Table 1:** Properties of B1 gypsum used as base material.

Purity (%)	Granulometry (mm)	Surface Hardness (Shore C)	Performance (kg/m^2^)/cm	Flexural Strength (N/mm^2^)	Compressive Strength (N/mm^2^)	Adherence (N/mm^2^)	pH
>75	0–1	≥45	10–12	≥2	≥ 2	>0.1	>6

**Table 2:** Composition of the gypsum mixes for the 12 specimens of 40 × 40 × 160 mm^3^.

Sample Series	Gypsum (g)	Water (g) or (mL)	W/G Ratio	Wood Shavings (g)	Sawdust (g)
**Reference**	4000	2200	0.55	-	-
**WS5**	4000	2200	0.55	200 (5%)	-
**WS10**	4000	2200	0.55	400 (10%)	-
**WS20**	4000	3200	0.80	800 (20%)	-
**S5**	4000	2200	0.55	-	200 (5%)
**S10**	4000	2200	0.55	-	400 (10%)
**S20**	4000	3200	0.80	-	800 (20%)

**Table 3:** Definition of a wetting–drying cycle.

Cycle	Relative Humidity (%)	Temperature (°C)	Time (h)
Wetting	90	23	24
Drying	30	55	24
Wetting	90	23	72
Drying	30	55	48

**Table 4:** Test results obtained for the composites after 0, 5, 10, and 15 wetting–drying cycles.

Samples	Density (g/cm^3^) (CoV, %)				Flexural Strength (MPa) (CoV, %)				Compressive Strength (MPa) (CoV, %)				Ultrasonic Velocity (m/s) (CoV, %)			
Cycles ^1^	0C	5C	10C	15C	0C	5C	10C	15C	0C	5C	10C	15C	0C	5C	10C	15C
**Reference**	1.28	1.22	1.17	1.14	3.48	3.28	2.35	2.14	8.53	8.03	5.60	4.05	2299	2259	1952	1810
	(2.77)	(2.84)	(2.81)	(2.85)	(5.60)	(5.47)	(5.56)	(5.68)	(5.71)	(5.49)	(5.75)	(5.43)	(2.88)	(2.93)	(2.82)	(2.95)
**WS5**	1.23	1.15	1.14	1.10	2.41	2.04	1.86	1.69	5.1	4.37	3.16	2.94	2187	1822	1633	1579
	(3.27)	(3.31)	(3.42)	(3.37)	(6.61)	(6.58)	(6.64)	(6.68)	(6.71)	(6.63)	(6.42)	(6.73)	(3.24)	(3.28)	(3.45)	(3.37)
**WS10**	1.20	1.14	1.13	1.09	2.06	1.63	1.59	1.51	4.18	3.57	2.89	2.56	2069	1497	1402	1360
	(4.08)	(4.10)	(4.11)	(4.16)	(8.23)	(8.19)	(8.37)	(8.26)	(8.29)	(8.41)	(8.33)	(8.17)	(4.12)	(4.03)	(4.14)	(4.09)
**WS20**	1.02	0.91	0.90	0.90	1.39	1.06	0.98	0.96	2.78	2.23	2.03	1.94	1652	1178	1128	1093
	(4.61)	(4.57)	(4.68)	(4.72)	(9.18)	(9.23)	(9.26)	(9.14)	(9.31)	(8.89)	(9.11)	(8.93)	(4.66)	(4.75)	(4.81)	(4.76)
**S5**	1.25	1.18	1.15	1.12	2.68	2.42	1.88	1.79	6.17	5.42	4.10	3.81	2239	1989	1896	1715
	(3.08)	(3.12)	(3.10)	(3.18)	(6.23)	(6.44)	(6.14)	(6.38)	(6.26)	(6.31)	(6.24)	(6.18)	(3.07)	(3.12)	(3.04)	(3.15)
**S10**	1.24	1.12	1.10	1.09	2.13	1.85	1.66	1.57	5.26	4.55	4.01	3.78	2101	1916	1749	1448
	(3.54)	(3.61)	(3.59)	(3.52)	(7.12)	(7.32)	(7.19)	(7.22)	(7.24)	(7.36)	(6.98)	(7.16)	(3.75)	(3.63)	(3.59)	(3.78)
**S20**	0.99	0.95	0.93	0.90	1.81	1.43	1.27	1.13	3.19	2.53	2.31	2.13	1784	1450	1199	1154
	(3.92)	(4.07)	(3.89)	(4.02)	(7.89)	(7.95)	(8.02)	(7.74)	(8.12)	(7.96)	(7.85)	(7.79)	(3.95)	(3.87)	(3.91)	(3.88)

^1^ 0C: 0 wetting–drying cycles; 5C: 5 cycles; 10C: 10 cycles; 15C: 15 cycles.

**Table 5:** Results of ANOVA for the flexural and compressive strength test results. Influence of wetting–drying cycles, type and percentage of wood waste added, and the interaction of both factors.

Control Factor		Sum of Square (SSA)	Degrees of Freedom (f)	Mean Square	FA0	*p*-Value
Flexural Strength	Wetting–Drying Cycles	6.908	3	2.303	128.922	4.07 × 10^−25^
	Wood Waste Addition (%)	22.160	6	3.693	206.771	2.46 × 10^−36^
	Interaction	1.809	18	0.101	5.629	2.89 × 10^−7^
	Total	31.875	83	-	-	-
Compressive Strength	Wetting–Drying Cycles	103.452	3	34.484	816.152	1.91 × 10^−88^
	Wood Waste Addition (%)	316.093	6	52.682	1246.855	7.57 × 10^−119^
	Interaction	41.063	18	2.281	53.993	8.22 × 10^−54^
	Total	466.524	167	-	-	-
